# Efficient decoy selection to improve virtual screening using machine learning models

**DOI:** 10.1186/s13321-025-01107-z

**Published:** 2025-10-30

**Authors:** Felipe Victoria-Muñoz, Janosch Menke, Norberto Sanchez-Cruz, Oliver Koch

**Affiliations:** 1 Institute of Pharmaceutical and Medicinal Chemistry, Universität Münster, Münster, Germany; 2 Department of Computer Science and Engineering, Chalmers University of Technology, Göteborg, Sweden; 3 Instituto de Química, Unidad Mérida, Universidad Nacional Autónoma de México, Mérida, México; 4 Center for Multiscale Theory and Computation, Universität Münster, Münster, Germany

**Keywords:** Specific scoring function, Protein-ligand interaction fingerprint, Molecular docking, Decoys, Virtual screening, PADIF

## Abstract

Machine learning models using protein-ligand interaction fingerprints show promise as target-specific scoring functions in drug discovery, but their performance critically depends on the underlying decoy selection strategies. Recognizing this critical role in model performance, various decoy selection strategies were analyzed to enhance machine learning models based on the Protein per Atom Score Contributions Derived Interaction Fingerprint (PADIF). We explored three distinct workflows for decoy selection: (1) random selection from extensive databases like ZINC15, (2) leveraging recurrent non-binders from high-throughput screening (HTS) assays stored as dark chemical matter, and (3) data augmentation by utilizing diverse conformations from docking results. Active molecules from ChEMBL, combined with these decoy approaches, were used to train and test different machine learning models based on PADIF. The final validation was done by confirming experimentally determined inactive compounds from the LIT-PCBA dataset. Our findings reveal that models trained with random selections from ZINC15 and compounds from dark chemical matter closely mimic the performance of those trained with actual non-binders, presenting viable alternatives for creating accurate models in the absence of specific inactivity data. Furthermore, all models showed an enhanced ability to explore new chemical spaces for their specific target and enhanced the top active compound selection over classical scoring functions, thereby boosting the screening power in molecular docking. These findings demonstrate that appropriate decoy selection strategies can maintain model accuracy while expanding applicability to targets even when lacking extensive experimental data.

## Scientific contribution

This study enhances the available options for decoys to create new bioactive machine learning classification models from public datasets. Through a molecular docking protocol and PADIF representation, we identify the benefits of using this methodology to improve binder selection in molecular docking

## Introduction

Molecular docking serves as a fundamental computational method in drug discovery. [1–3]. It enables scientists to quickly and reliably produce and evaluate potential binding conformations of a small molecule to a given (protein) target. One vital component in molecular docking is the scoring function, serving to rank the various binding conformations (poses) produced during the docking process. [4]. As the scoring functions only offer an estimate of fit, numerous different scoring functions have been developed over recent decades, with each approach offering distinct advantages but also having specific limitations [5, 6]. As these scoring functions only provide a rough estimate of how well a given ligand binds, a thorough analysis of calculated poses is needed.[7] For this, researchers tend to rely on integrating existing structural information into the decision-making process when selecting which ligands to advance in their current drug discovery campaign. One such approach involves the use of existing information from known protein-ligand complexes. An experienced drug designer is using such information by analyzing the poses via visual inspection.

However, efforts have been made to automate this process and one such approach involves protein-ligand interaction fingerprints. An early example of such fingerprint is the structural interaction fingerprint (SIFt) [8]. Here, the interaction patterns of known protein-ligand complex structures are compared to the interaction of poses obtained from docking to enhance the performance during post-processing. Since then, the availability of large amounts of data (the area of “big data”) and developments in the field of machine learning (ML) have significantly advanced this area. This has made ML-based scoring increasingly important for addressing docking limitations [9, 10]. One of the first was RF-Score developed by Ballester and Mitchell [11], which captures the features of protein and ligand interactions using pairs of atoms and trains random forest (RF) models [11]. Here, the combination of the protein-ligand interaction fingerprint (PLIF) representation and machine learning models was used to improve original rule-based scoring functions. Sánchez-Cruz et al. [12] used Extended Connectivity Interaction Features to encode each atom in a protein-ligand complex, providing a comprehensive description of all atoms involved in the binding. With these fingerprints, two ML models were trained for affinity ($pK_d$/$pK_i$) prediction [12], outperforming eight alternative docking scoring functions. By expanding the concept of PLIFs further, the approach of protein-ligand extended connectivity utilizes extended connectivity fingerprints to map out the structural details of ligands and residues, focusing on the interaction depth or radius among atoms. This approach achieved a correlation above 0.8 on the PDBind when comparing predicted and experimental data [13]. In a different approach, the SMPLIP-Score merges interaction fingerprints with molecular substructures, creating a novel model representation. This technique has been shown to significantly boost the predictive accuracy of affinity predictions, outperforming several scoring functions assessed during CASF-2016 [14].

In the described examples, the ML models were employed to enhance the *“scoring power”* (affinity prediction) associated with scoring functions. However, in virtual screening campaigns, the *“screening power”* is the most critical parameter. Screening power refers to the ability to select ligands in a mix of binders and non-binders, where the correct selection of ligands could lead to the discovery of molecules with biological activity [15, 16]. In these scenarios, our PADIF fingerprint (protein per atom score contributions derived interaction fingerprint) shows a superior ability to retrieve ligands from datasets containing active and decoy compounds, when compared with other scoring functions and PLIFs (ChemPLP, IFP, and TIFP) [17]. Later, we used PADIF fingerprints to train ML models for target prediction or inverse screening approaches: Models were trained for 20 different target datasets with bioactivity data retrieved from ChEMBL [18]. These models demonstrated the ability to accurately select the target for active molecules from a mix of targets, circumventing the inter-protein scoring noise that normally prevents docking from being used for target selection. [18]. Nonetheless, the selection for some groups of targets was incorrect, indicating confusion in the models due to incorrect selection from the decoy sets.

In this work, the decoy selection, the act of choosing inactive or non-binding compounds that resemble active compounds in their physicochemical properties but lack biological activity, was performed using a cutoff activity value for the bioactivity data retrieved from ChEMBL. This leads to issues associated with the incorrect representation of negative interactions. The cut-off-based approach is widely used in bioactivity prediction. The drawback with this approach is the introduction of bias contained within the databases, which usually feature more binders ($\le 10 \mu M$) than non-binders [19–21]. Alternative examples have focused on sampling random molecules as decoys, which positively impacts model performance but increases the presence of false negatives in compound predictions [22, 23]. Given these considerations, the existing decoy datasets exhibit inherent biases [24], necessitating the exploration of more effective methods for enhancing bioactivity classifiers.

## Results and Discussion

Nine protein targets were selected based on three key criteria: numbers of active compounds, availability of confirmed non-binders, and accessible high-quality protein structures in public databases. The selected targets include both enzymes and receptors, representing the most common target classes in medicinal chemistry. Importantly, these targets exhibit varying numbers of active compounds, creating diverse dataset scenarios that allow comprehensive testing of our computational approach across different data abundance conditions. (see Table 1).

**Table 1:** Information related to targets used in this study. The number of actives are compounds that comply with all filters. The number of decoys is lower than the number of true non-binders, since $10\%$ of the actives were separated for the external validation set

Target name	ID	ChEMBL ID	PDB ID	Ligand ID	Number of actives	Number of True non-binders	Number of Decoys
Aldehyde Dehydrogenase 1	ALDH1	CHEMBL 3577	4W7P	3J7	245	980	882
FLAP Endonuclease	FEN1	CHEMBL 5027	5FV7	R3Z	61	244	220
Glucocere- brosidase	GBA	CHEMBL 2179	2XWE	AMF	307	1228	1105
Isocitrate Dehydrogenase	IDH1	CHEMBL 2007625	5SVF	70P	1860	7440	6696
Mitogen-activated protein kinase 1	MAPK1	CHEMBL 4040	1PME	SB2	3906	15624	14062
Mechanistic target of rapamycin	MTORC1	CHEMBL 2842	1NSG	RAD	4753	19012	17111
Pyruvate kinase muscle isoform 2	PKM2	CHEMBL 1075189	3ME3	3SZ	86	344	310
Peroxisome proliferator-activated receptor gamma	PPARG	CHEMBL 235	1ZGY	BRL	4298	17192	15473
Vitamin D receptor	VDR	CHEMBL 1977	3A2J	TEJ	459	1836	1652

**Fig. 1:**
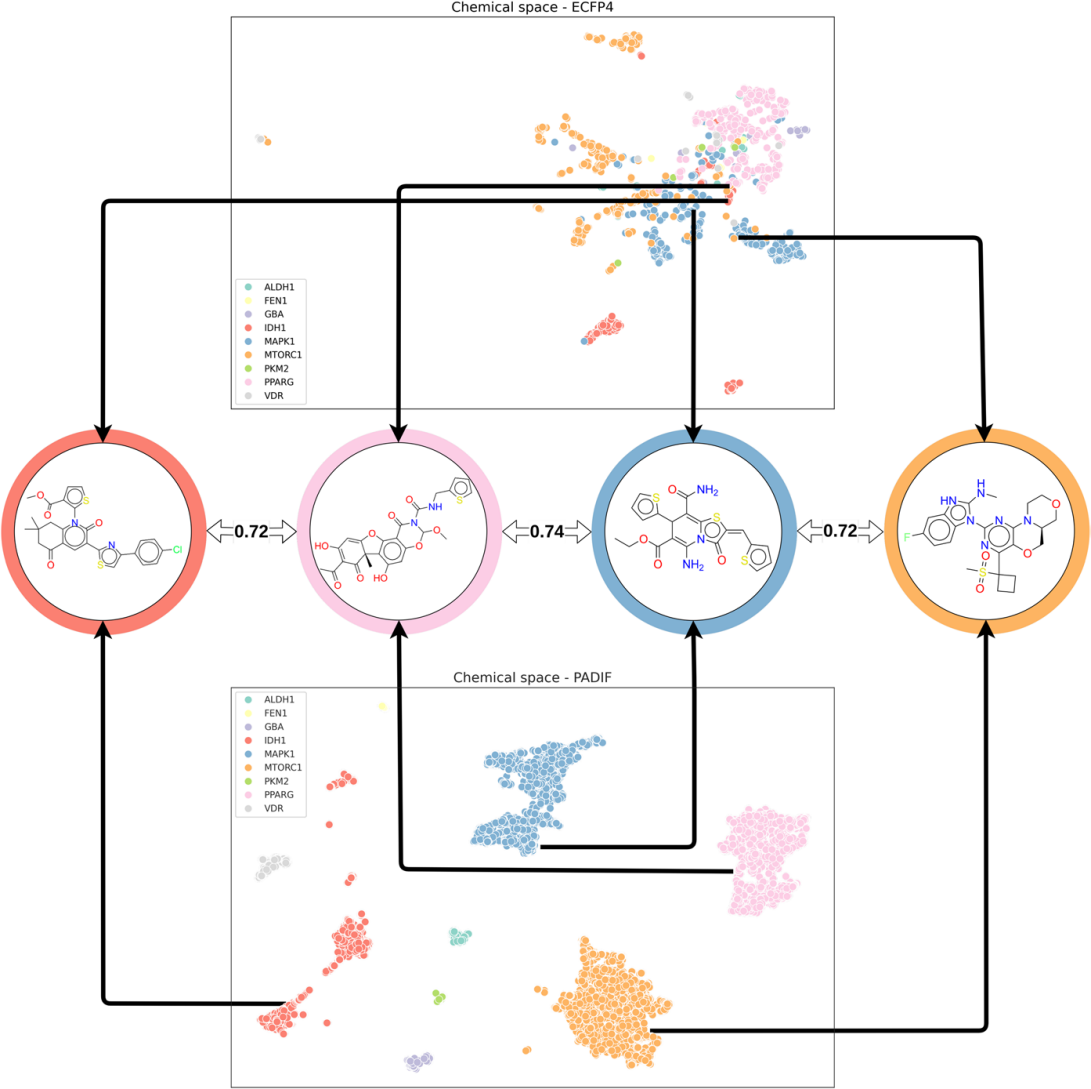
Chemical space visualization of active compounds by comparing Morgan and $PADIF_{STD}$ representations. The Tanimoto similarity values based on Morgan fingerprints are added between the example molecules

Molecules from the datasets were visualized in chemical space using the Morgan fingerprint (radius: 2, length: 2048) and the $PADIF_{STD}$ using the UMAP dimensionality reduction [25] (see Fig. 1). Interestingly, classifying molecules by their activities using the Morgan fingerprint proves to be a challenging task, since the interclass similarities across all target sets exceeded 0.63. However, when examining the plot using our PADIF, one can see a strong separation between the different protein targets. As the PADIF represents the chemical space through interactions rather than the structural patterns of molecules, it captures functionally relevant similarities across diverse chemical scaffolds, which traditional fingerprints cannot do. This highlights PADIF as efficient in differentiating active compounds and enables robust classification using similarity metrics across all targets regardless of the structural heterogeneity of active compounds or protein binding sites.

In the Jasper et al. [17] paper, PADIF was compared to other protein-ligand interaction fingerprints like IFP and TIFP [17]. These methods often fall short because they primarily focus on classifying the existence of a contact between the protein and ligand, rather than the specific nature and strength of that interaction. PADIF differentiates itself by using a more granular approach. It classifies atoms into several distinct types, such as donor, acceptor, nonpolar, metal, and charged, and then uses a piecewise linear potential to assign a numerical value to each specific interaction type. This allows PADIF to capture a richer and more nuanced representation of the binding interface, which can lead to better performance in virtual screening tasks compared to fingerprints that simply register the presence or absence of a contact.

**Fig. 2:**
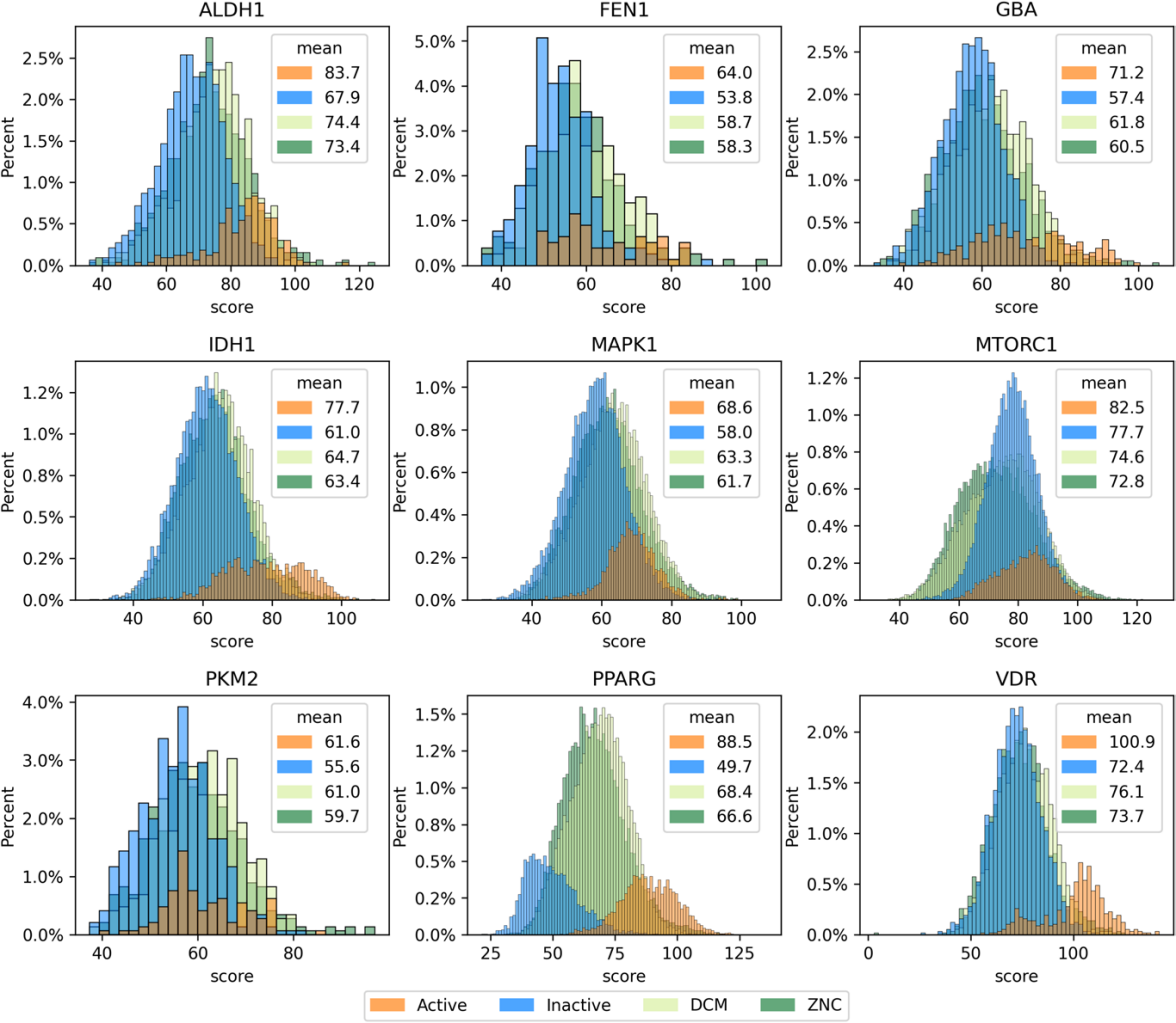
Histogram plot between ChemPLP score (score) and total percentage of molecules in the target sets. The legend includes the mean of ChemPLP score by category, where orange, blue, light green, and dark green represent actives, true inactives, DCM, and ZNC decoys

Another important point is the analysis of the score distributions between actives and inactives/decoys. Figure 2 compares the ChemPLP score distribution across the different categories (actives, inactives, DCM, ZNC). The DIV decoys are excluded from this analysis because these are the identical active molecules with a defined lower score and different *‘wrong’* binding conformation. As the figure highlights, an overlap of the score distributions between actives and inactives/decoys is occurring which prevents discrimination purely based on ChemPLP scoring. Our approach to design the target datasets was to replicate the inherent imbalance found in experimental testing. In traditional in-vitro assays, the number of active compounds is often very small when compared to the vast number of inactive ones. [26, 27]. Notably, PPARG had fewer non-binders, a result of the filtering criteria applied during dataset building and the lower availability of inactive molecules in LIT-PCBA [28].

After docking and conformational sampling, protein-ligand interaction fingerprints (PADIFs) were generated for each molecule. The datasets were subsequently split into training, test and external validation sets using random, scaffold, and fingerprint-based strategies to promote robustness and minimize data leakage. Machine learning models were trained on these datasets and validated against a test set of true binders/non-binders, aiming to identify the most effective decoy approach that behaves similarly to actual non-binders.

**Fig. 3:**
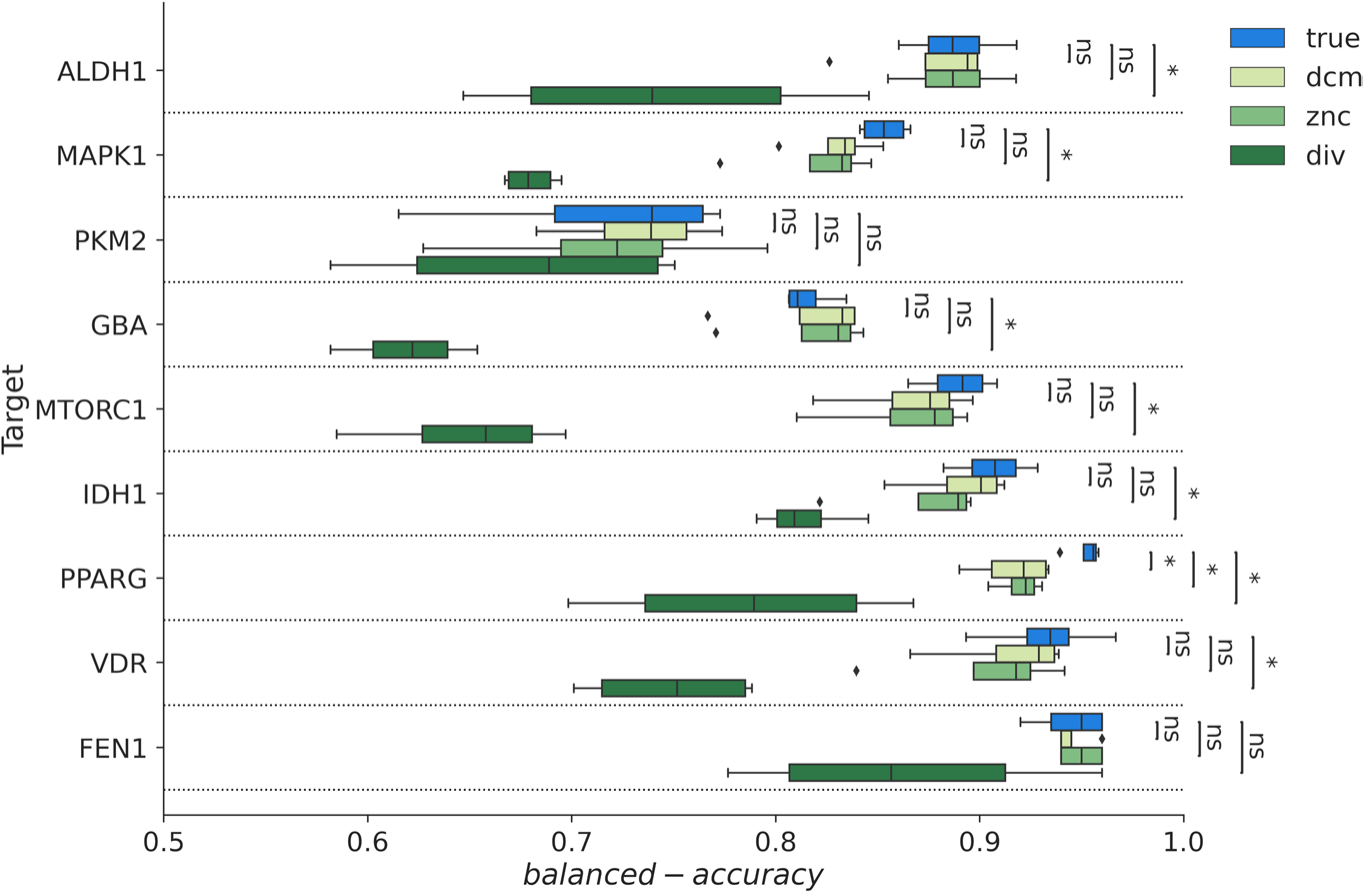
Box plot visualization of the Balanced Accuracy (*BA*) for each model (SVM, RF, XGBoost, MLP). The depiction uses light green, green, and dark green to denote the BA for the DCM, ZNC, and diverse solution sets, while the true dataset is marked in blue. Statistical difference between Balanced accuracy into each model was calculated with an independent t-test. (ns: $5.00e^{-02}< p <= 1.00e^{+00}$ / *: $1.00e^{-02}< p <= 5.00e^{-02}$ / **: $1.00e^{-03}< p <= 1.00e^{-02}$ / ***: $1.00e^{-04}< p <= 1.00e^{-03}$)

Figure 3 displays the balanced accuracy (BA) for various sets based on random split. Across all targets, the mean BA exceeds 0.8 in true models, except for PKM2, indicating PADIF’s ability to create ML models with strong screening power. DCM and ZNC decoy sets produced balanced accuracy values comparable to models validated against true inactive compounds, indicating these methods generate realistic negative examples. In contrast, DIV models showed the highest variability and lowest average performance across targets. This was to be expected, as the various diverse solutions still show an overlap in their PADIFs. Despite this, we can see that valid models can still be generated with minimal computer effort. However, for ALDH1, FEN1, IDH1, VDR, and PPARG, the mean BA of DIV models exceeds 0.7.

Using the alternate fingerprint-based and scaffold-based splitting methods, we can see an expected change in the absolute performance, but the above-described behavior and trends remain the same, confirming the consistency of these findings across different validation strategies.(Figures S1 and S2 in the Supporting Information).

**Fig. 4:**
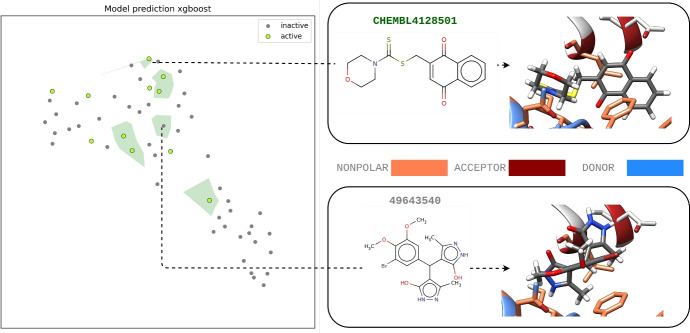
Visualization of the chemical space and protein interactions for the PKM2 test set. The chemical space representation uses PADIF for active and inactive compounds, with a green background denoting the active decision boundary by the XGBoost model is in the left side. Protein atoms are color-coded to show nonpolar, acceptor, and donor atoms according to PADIF positive interactions

To illustrate a specific challenge with PKM2, Fig. 4 demonstrates the model’s prediction for the true test set built with XGBoost using active and true inactive molecules. In this visualization, active and inactive molecules are marked by dots, with the green background indicating the region where the model predicts compounds as active (decision boundary). The figure reveals that active and inactive compounds occupy similar regions in chemical space, particularly those with high PADIF similarity scores, which creates significant classification difficulty. The false positive classified by the model (CHEMBL49643540) showed a high mean cosine similarity (0.74) of PADIF fingerprint with all actives in this dataset. Despite low structural similarity to CHEMBL4128501, its high PADIF similarity explains the model’s confusion. This classification challenge can be attributed to PKM2’s binding site characteristics. The binding site is located on the protein’s exterior surface, which limits the diversity of amino acid residues available for molecular interactions. This constraint leads to similar interaction patterns between active and inactive molecules, patterns that are captured by the PADIF fingerprint and consequently make them difficult for machine learning models to distinguish.

**Fig. 5:**
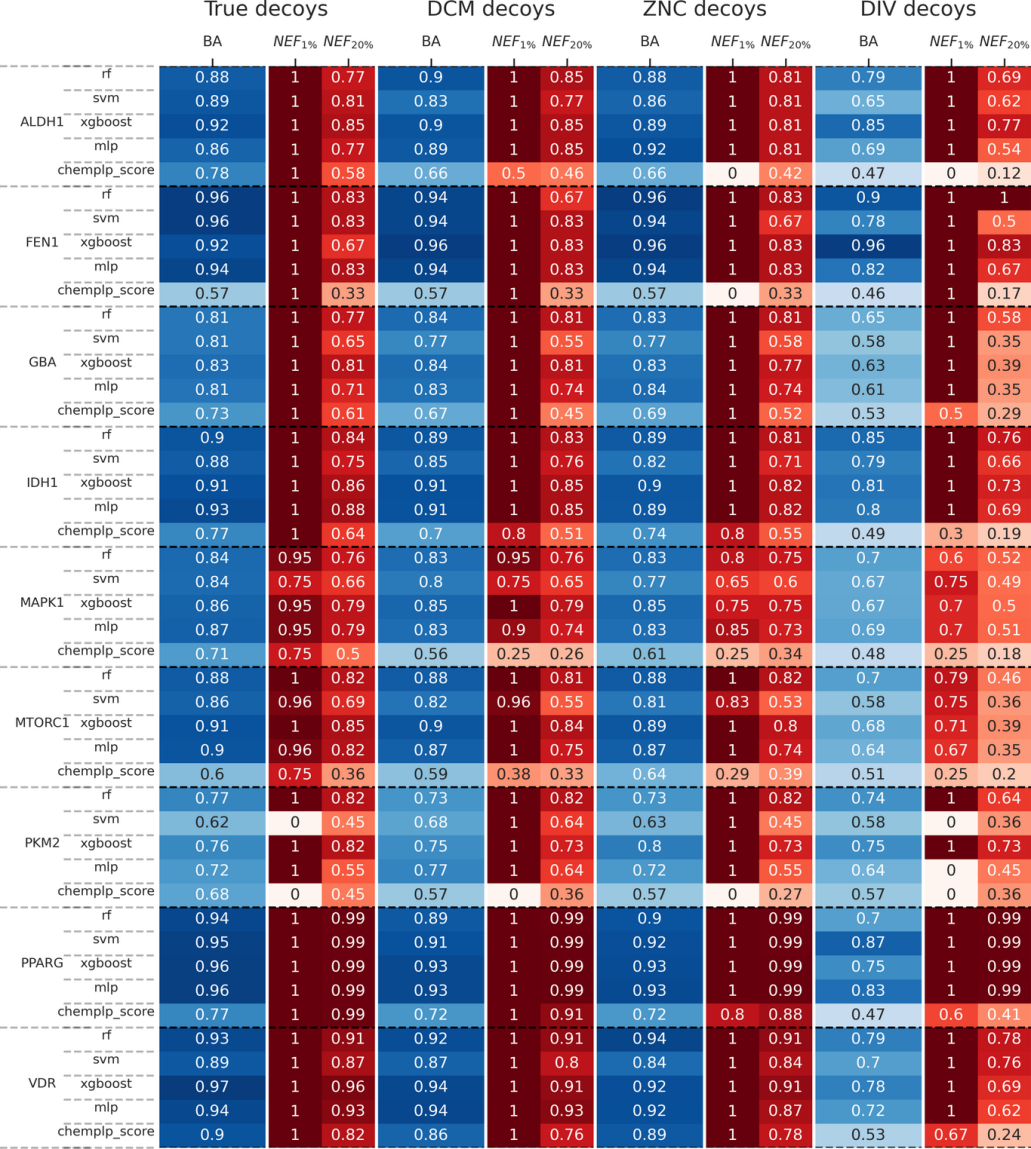
Heatmap displaying balanced accuracy (*BA*) and Normalized Enrichment Factors ($NEF_{1\%}$ and $NEF_{20\%}$) for each model used (RF, SVM, XGBoost, MLP) and the ChemPLP score for each target across all decoy types in the random splitting

Besides model performance, we also investigated the virtual screening power by examining the normalized enrichment factor (*NEF*), which serves as a vital metric in the rapid selection of actives in virtual screening. *NEF* measures the proportion of actives accurately classified at a specified percentage [29, 30]. The results in Fig. 5 show that, especially for PKM2, the improvement is noticeable since active, inactive, and decoy compounds share similar ChemPLP scores, complicating the selection based solely on this scoring. For the other targets, the $NEF_{20}\%$ reveals that all models and most datasets see improvement.

PPARG and VDR already exhibit good $NEF_1\%$ and $NEF_{20}\%$ based solely on the ChemPLP score. But this is explainable, since the true actives and inactives can already be separated by the ChemPLP score (see figure 2). However, this apparent success should be interpreted cautiously, as the significant molecular weight differences between active and inactive compounds allow ChemPLP to easily distinguish the two classes through this bias rather than genuine binding affinity prediction (see Supporting Information Figure S3). Conversely, PKM2 models demonstrate a superior ability to select active compounds at the top of the ranking, doubling the efficacy in comparison to the scoring function. This behavior can be attributed to the model’s capacity to discern critical interactions for active compounds. SVM models, in comparison, showed lower *NEF* across all types of decoys for PKM2, having the lowest values for BA in all models

**Fig. 6:**
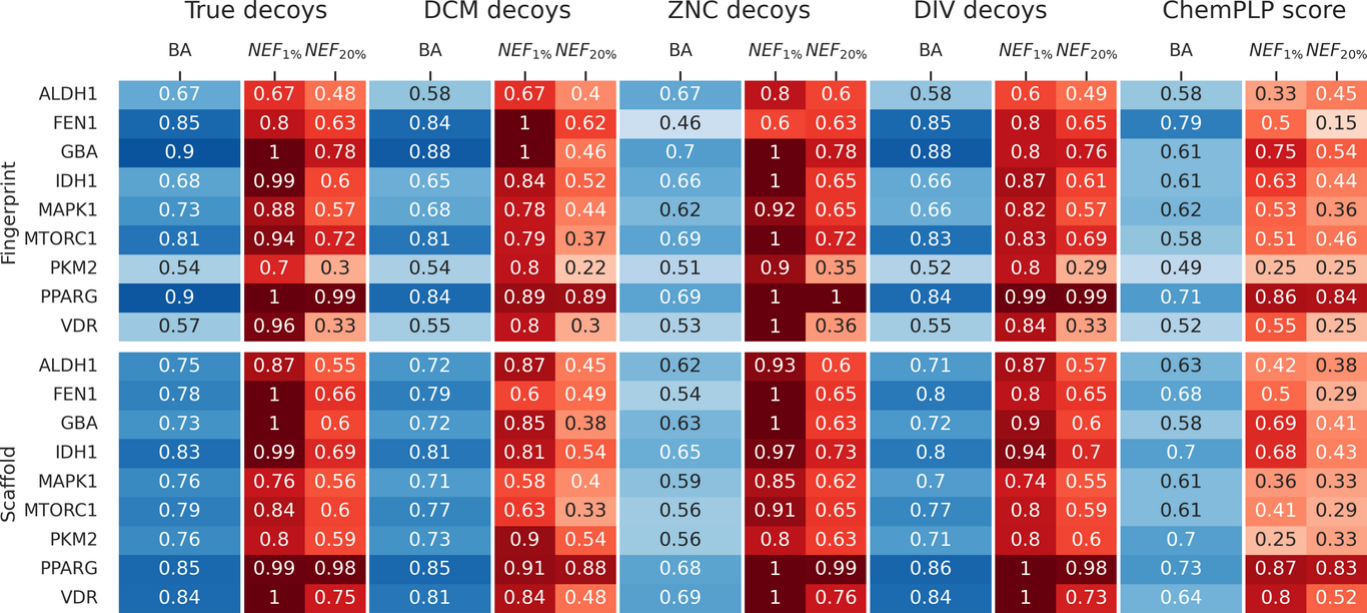
Analysis of mean Balanced Accuracy (*BA*) and Normalized Enrichment Factors ($NEF_{1\%}$ and $NEF_{20\%}$), fingerprint and scaffold split, showcasing performance across each decoy option and the ChemPLP score

Based on these metrics, random decoy selection (DCM, ZNC) performed better than diverse pose-based selections (DIV). Nonetheless, all options demonstrated an improvement in active selection compared to the ChemPLP score alone. DCM and ZNC models behaved similarly when tested in randomly split sets (Fig. 5). However, when employing fingerprint and scaffold splitters to maximize structural differences between test and train sets, as shown in Fig. 6, ZNC decoys exhibited the lowest *BA* values, while DCM decoys performed comparably to models trained with real inactive compounds. Nevertheless, the $NEF_1\%$ and $NEF_{20}\%$ values in models trained with ZNC decoys were superior to those trained with DCM decoys, which can be attributed to the greater structural diversity in the ZNC database, providing sufficient structural variation to improve the training of bioactive classification tools. Most importantly, these models exhibit potential for extrapolating to previously unseen chemical spaces, as demonstrated by the fingerprint-based and scaffold-based splitting tests. This is crucial, given that one of the goals of this fingerprint is to identify molecules dissimilar from known active compounds with a similar interaction pattern. This fits the basic idea of the PADIF approach which completely neglects all structural ligand information.[17] The fingerprint is only based on the protein atom contributions of the underlying scoring function. The ligand atom contributions are ignored, which makes the models independent of the underlying ligand structure and focus solely on the interaction to the protein.

The DIV approach also shows a significant increase in comparison to the ChemPLP score. This is interesting, since only a small number of actives is suitable to create a model based on diverse poses which leads to an improvement in virtual screening performance for the next round. It could be expected that modifying the parameter for creating the diverse solutions as future work can further improve the overall performance.

**Fig. 7:**
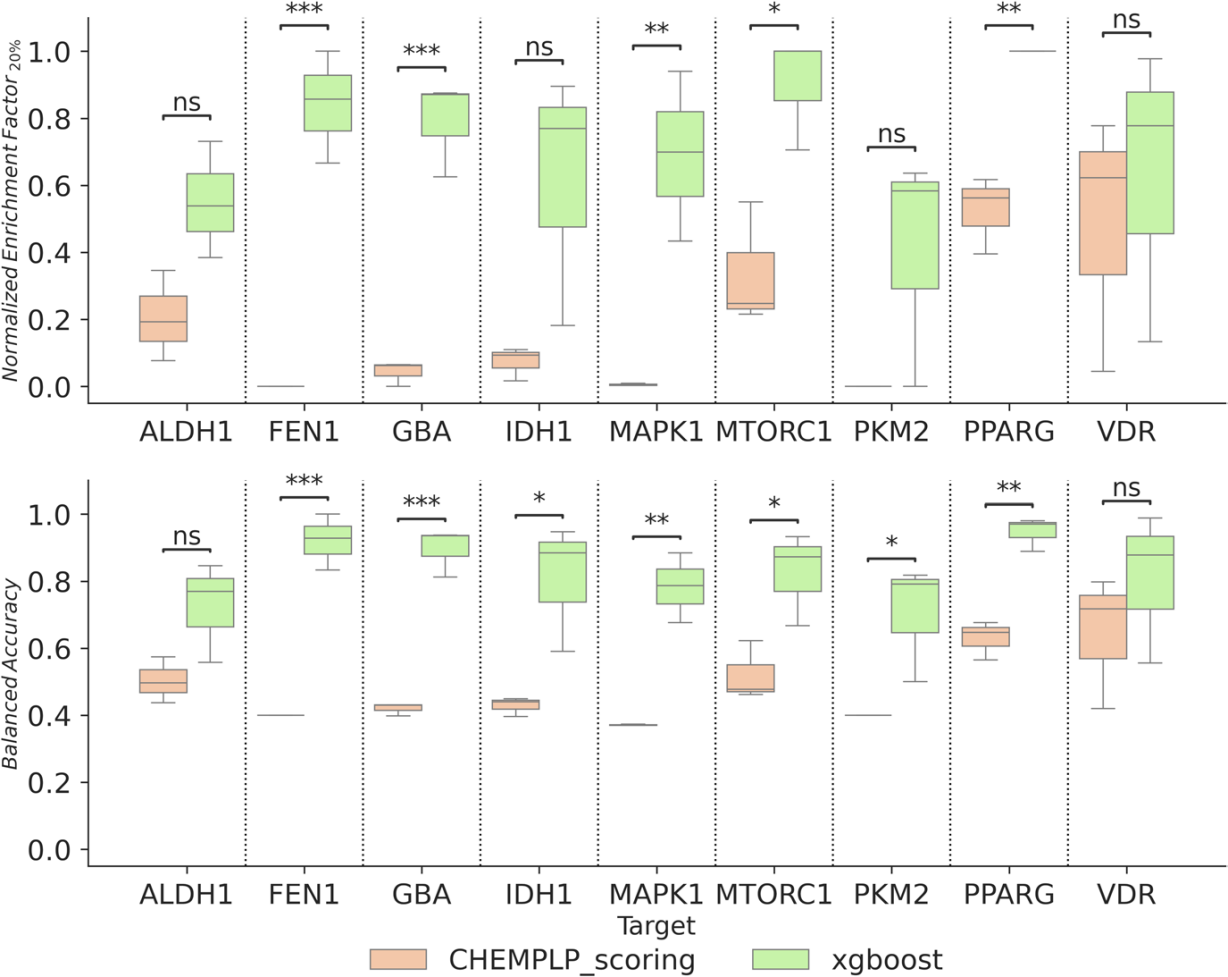
$NEF_{20\%}$ and *BA* for multitarget test consisting of True and DCM models, combining results from all test sets (random, fingerprint, scaffold). Statistical difference between ChemPLP scoring and XGBoost were calculated with independent t-test (ns: $5.00e^{-02}< p <= 1.00e^{+00}$ / *: $1.00e^{-02}< p <= 5.00e^{-02}$ / **: $1.00e^{-03}< p <= 1.00e^{-02}$ / ***: $1.00e^{-04}< p <= 1.00e^{-03}$)

As a final target-prediction and ligand profiling performance test, we focused solely on all actives from all targets combined into one dataset. This dataset was docked into all targets with the aim of identifying the real target for each active compounds. General scoring functions fail due to the known inter-protein scoring noise.[18, 31] Fig. 7 shows the $NEF_{20}\%$ and *BA* displayed for each target between ChemPLP scoring and XGBoost model. Both metrics show that this model can accurately select all the actives present in a mix of real binders for these targets, as the $NEF_{20}\%$ demonstrates the top selection of these compounds and *BA* the active selection over the decoy ones. Only for ALDH1 and VDR targets the p-value did not reveal statistically significant differences between both metrics. Thereby highlighting the enhanced capacity of these ML models to improve the ligand profiling and yield better results in target selection tasks. This was also shown previously by Nogueira and Koch [18]. Correct classification is likely attributable to the PADIF representation’s ability to distinctly categorize similar active compounds based on molecular interactions rather than structure alone, as is shown in Fig. 1.

**Fig. 8:**
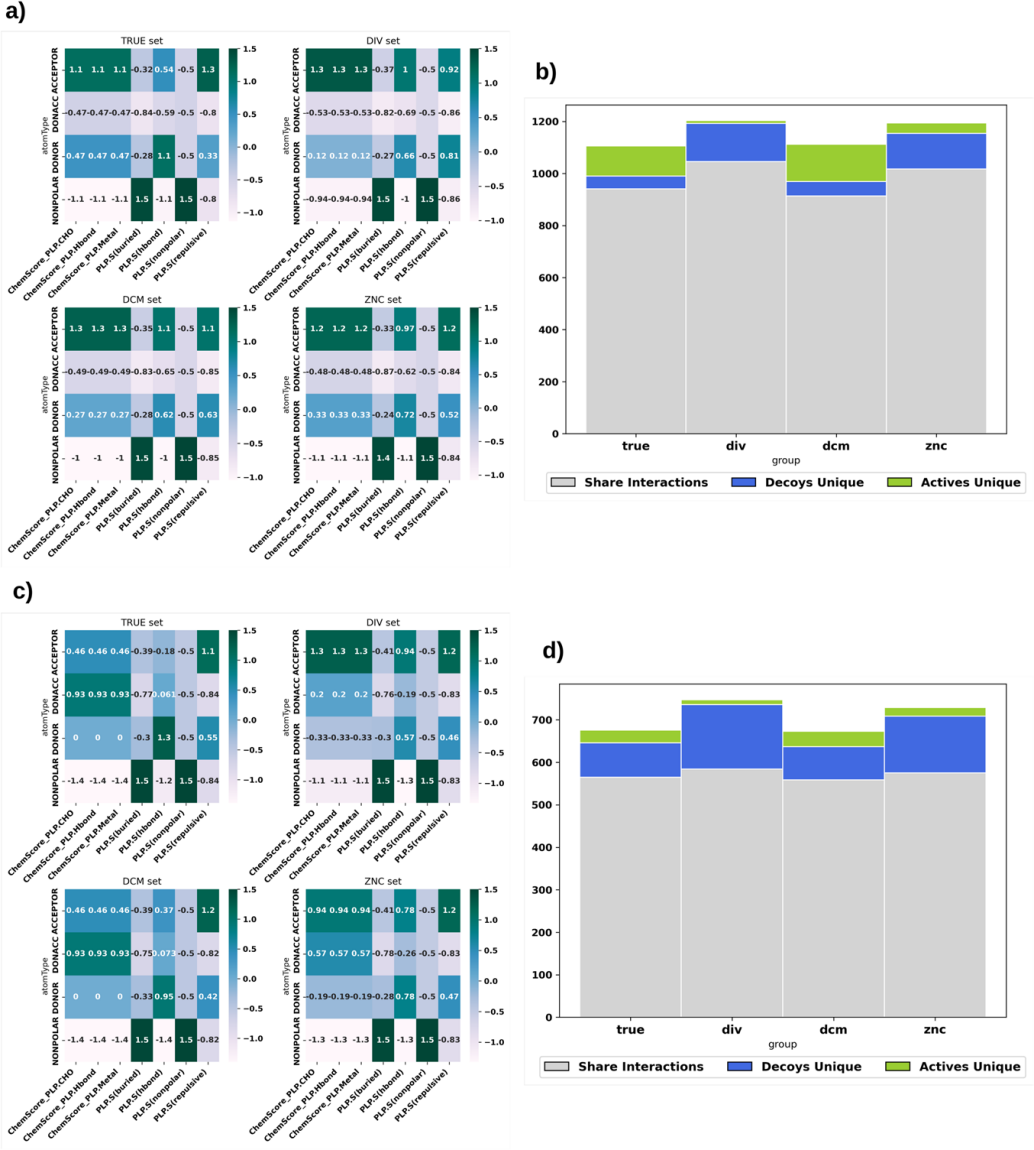
Interaction analysis for MTORC1 and VDR, illustrating mean interaction terms by atom type (a, b) and unique interactions for actives and decoys compounds (c, d)

After analyzing the performance of models trained with various decoy strategies, it becomes evident that each approach yields distinct interaction patterns, with DIV decoys showing the most significant deviation from true inactive compounds. Figure 8a and d illustrate the frequency of interactions by atom type for each decoy alternative, revealing systematic differences between decoy methods.

For the MTORC1 target, the primary distinction between true inactives and DCM decoys lies in ChemScore PLP interactions with DONOR atoms, while DIV decoys exhibit notably fewer occurrences of this interaction type. Similarly, VDR shows substantial changes in ChemScore PLP interactions and altered distributions for ACCEPTOR, DONOR, and DONACC atoms. These differences may reflect the inherent challenge of generating conformationally diverse decoys that differ sufficiently from active compounds.

## Methods

### Dataset building

Active compounds were retrieved from ChEMBL33, and true non-binders (TRUE) were selected from the LIT-PCBA datasets [28, 32], whereas only true non-binders that are dissimilar to the active compounds were kept. Although recently, some problems as data leakage and molecular redundancy with the LIT-PCBA dataset were reported [33], this is not relevant for our work. We focused only on the inactive compounds and checked for similarity to our active compounds. Unique molecules with a molecular weight between 180 and 900 Da, activity types $IC_{50}$, $EC_{50}$, $K_i$, $K_d$, confidence classes of 8 or 9, and activity lower than $10 \mu M$ were identified as actives compounds. Besides the true inactives from the LIT-PCBA datasets, decoys were selected through three distinct approaches: a random selection from the ZINC15 database (ZNC), from the Dark Chemical Matter (DCM) dataset [34, 35] and using diverse binding conformations (DIV) of active compounds. To ensure dataset balance and biological relevance, a 4:1 ratio of decoys to active compounds was used for each target for the True Inactives, ZNC and DCM datasets.

The ZNC strategy mimics the conventional method of selecting decoys for bioactivity prediction, while the DCM strategy comprises molecules characterized by their lack of biological activity identified through multiple high-throughput screening campaigns across several targets [35], which makes them ideal decoys. The last strategy represents a data augmentation strategy, where multiple distinct conformations per molecule were generated using the diverse solutions option from the GOLD docking package (DIV). This option forces GOLD to systematically generate conformational variants that represents significant interaction differences from the original binding pose (see docking section). The idea was to avoid any decoy selection strategy and only work with the active ones. This would also mean a massive reduction in the computer resources required, as otherwise the decoys would also have to be docked. The various solutions can be generated directly in the docking run of the active ones.

### Molecule preparation, and molecular docking

Proteins and small molecules (active, decoys and co-crystal compounds) were prepared using the CCDC Python API (v3.0.1). For protein files, the ligand, water molecules, redundant chains, and ions were removed; hydrogens were added under pH 7.4 conditions. Small molecules, represented in SMILES notation, were minimized, protonated, and bond types standardized according to the GOLD docking protocol. All structural files were saved as mol2 files for the docking process. Docking processes were conducted using the CCDC Python API. ChemPLP was selected as the fitness function with a search efficiency of $200\%$, while early termination was disabled. Options to flip amide bonds, pyramidal nitrogen, save per atom scores, and concatenated output were enabled, requiring 20 different solutions per docking.

To ensure the robustness of our methodology, we carefully selected our protein files by cross-referencing with existing ChEMBL data. Our selection criteria prioritized structures with high resolution and the presence of a co-crystallized ligand. These complexes were crucial for validating our docking protocol. We performed a redocking protocol, where the known co-crystallized ligands were docked back into their respective binding sites. This allowed us to systematically optimize key docking parameters, such as the position and size of the binding box and the search efficiency. The conditions that yielded the lowest Root Mean Square Deviation (RMSD) between the redocked pose and the crystallographic conformation were selected for all subsequent virtual screenings.

For the diverse solution dataset, the diverse solution option was enabled with a minimum RMSD of 1.5 Å, leading to conformation cluster with a 1.5 difference between each conformation. The binding site was determined by the ligand’s location in the original complex. Furthermore, some molecules may fail the preparation process and are consequently ignored during the molecular docking workflow; this data is detailed in the Supporting Information, (Table S2: Actives per Train, Test sets and total used for each target).

### PADIF extraction

We re-implemented the original PADIF calculation in Python. According to the original publication, PADIF is defined by the ChemPLP contributions or interaction terms (ChemScore PLP.Hbond, ChemScore PLP.CHO, ChemScore PLP.Metal, PLP.S(bond), PLP.S(metal), PLP.S(buried), PLP.S(nonpolar), PLP.S(repulsive)). Thus, in PADIF, the signs of the first three interaction terms were inverted, standardizing the sign of all terms. Jasper et al. [17] provide an in-depth explanation of this modification and other details [17]. This reimplementation was performed in Python and is available at https://github.com/kochgroup/PADIF-wf.

**Fig. 9:**
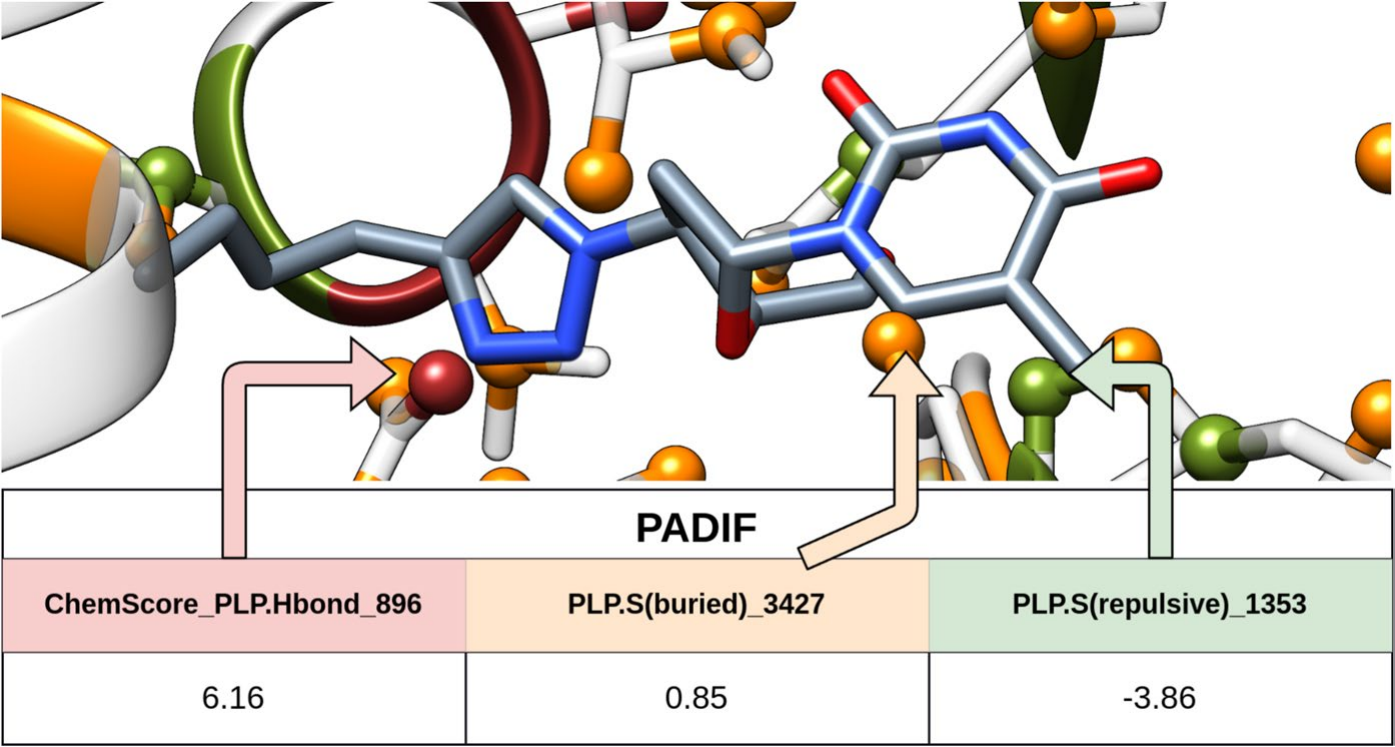
Graphical explanation of PADIF (protein per atom score contributions derived interaction fingerprint)

For each docking solution, a PADIF (see Fig. 9) was generated using the best conformation for actives based on the highest ChemPLP score, as well as molecules from the LIT-PCBA (TRUE set), and the decoy sets ZNC and DCM.[17, 28, 34, 35]. The DIV set was built with random conformations from the same docking results (excluding the best conformation). To ensure diversity, the diverse solutions option was enabled, with parameters set to produce conformations that had an RMSD larger than 1.5 Å and were grouped into a single cluster.

The PADIF fingerprint used for creating the ML models depends on the binding site size and the number of involved residues. Therefore, the PADIFs of different targets are not comparable. To represent a standard PADIF across all targets, the PADIF obtained for each molecule was characterized by a combination of residue, type of interaction, and type of atom (named $PADIF_{STD}$). The value assigned was the mean value of all protein atoms involved in these three characteristics, resulting in a maximum of 800 keys per molecule (20 residues, 8 types of interaction, 5 types of atoms).

### Train, test, and validation datasets

**Fig. 10:**
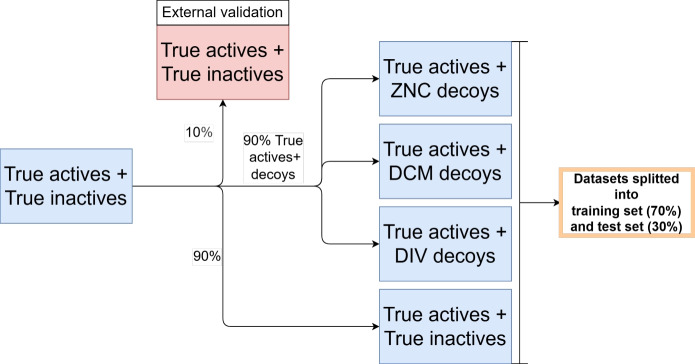
Workflow followed for splitting, creating, and testing the models from the datasets. For each target, four datasets were created. The first was a "true set," containing actual active and inactive molecules. The other three were "decoy sets," each containing the true active molecules mixed with artificially generated decoy molecules. To begin, $10\%$ of the true set was separated and used as an external validation set. The remaining $90\%$ of the active molecules from the true set were then used to create the three decoy datasets

Overall, four datasets were created based on the available true actives (see Fig. 10). First of all, the initial dataset of actives was enriched with true inactive compounds. This initial dataset was first divided into two parts for each target. $10\%$ of the true actives and true inactives were separated to serve as an external validation dataset for calculating the final quality metrics of the models. The remaining $90\%$ of the data represents dataset 1. The true actives of this dataset were then used to create further three new datasets: They were enriched with decoys from ZINC (dataset 2), decoys from DCM (dataset 3) and diverse solutions (dataset 4). The four retrieved datasets were subsequently split for model training and testing: 70 % of the data was designated as the training set, while the remaining 30% was used as the test set. This entire process was carried out on a single file that was processed directly using PyCaret library [36]. Random, scaffold, and fingerprint splitters were utilized to ensure diverse molecular representation across the sets, facilitating the exploration of new chemical spaces. Scaffold and fingerprint split are selections based on molecular structures, where the Murcko scaffold is used for the former and the Morgan fingerprint with a radius of 2 for the latter. The splitting was performed using Scikit-learn (v.1.1.3) and DeepChem (v.2.6.1) [37, 38].

### Model creation

A variety of machine learning models were trained to more thoroughly assess the different splitting strategies. These different models were based on: Random forests (RF) [39], support vector machines (SVM) [40], extreme gradient boosting (XGBoost) [41], and multi-layer perceptrons (MLP) [42]. All models were implemented using the PyCaret library (v.3.0.0) [43]. The model choice was based on preliminary studies conducted with PADIF (SVM, MLP) and other common learning strategies used in the literature (RF, XGBoost). The data underwent z-score normalization for feature standardization, stratified k-fold cross-validation for fold generation, and the Adaptive Synthetic Sampling (ADASYN) based correction for class imbalance, that creates synthetic datapoints for minority class [44–46]. Additionally, we mitigated the risk of overfitting by enabling an early stopping mechanism for the MLP models, where the training is prematurely ended when the validation loss does not improve. This mechanism ensures that our models remain capable of generalizing well to new data points.

### Statistics and quality metrics

The *F*1 score was chosen as the metric to guide the hyperparameter search and optimization during training, combining precision (the fraction of correctly classified actives among all retrieved) and recall (the fraction of correctly classified actives among all actual actives) [47, 48]. Additional metrics include the balanced accuracy, which offers insight into prediction accuracy with a focus on the active class, which is the minority class [41].

1 $$\begin{aligned} & F1 = \frac{TP}{TP + \frac{1}{2}(FP + FN)} \end{aligned}$$

2 $$\begin{aligned} & \text {Balanced accuracy} = \frac{1}{2} ( \frac{TP}{P} + \frac{TN}{N}) \end{aligned}$$

Where TP, FP, and FN represent the true positives, false positives, and false negatives in the classification, respectively. P and N denote the total positives and negatives.

The enrichment factor (*EF*) is another important metric to consider. It is used to measure the improvement in the first selection of active molecules, highlighting the top selections at determined percentages in the retrieval list for virtual screening workflows [29, 30].

3 $$\begin{aligned} EF_{\text {x\%}} = \frac{N_{\text {active x\%}} / N_{\text {total x\%}}}{N_{\text {actives}} / N_{\text {total}}} \end{aligned}$$

To have a better understanding of EF and avoid the proportion of active compounds in datasets, normalized enrichment factors were used [49]. For this, EF values were divided by the maximum possible value (EF(max)x%) at this specific percentage, rearranging between 0 to 1. $NEF_1\%$ and $NEF_{20}\%$ were used to compare developed models. Where $1\%$ is the most common value reported in virtual screenings [49], and $20\%$ represents the proportion of actives in these databases.

4 $$\begin{aligned} NEF_{\text {x\%}} = \frac{EF_{\text {x\%}}}{EF(max)_{\text {x\%}}} \end{aligned}$$

## Conclusion

In this study, we investigated various decoy selection strategies to construct precise activity models utilizing our PADIF approach. The basis builds true actives from ChEMBL and true inactives from the LIT-PCBA dataset, whereas decoys were selected from ZINC15 and the dark chemical matter dataset. A further approach using the diverse solution option of GOLD was also analyzed. Subsequently, different molecular splitters were also applied to assess our model’s ability to navigate and identify novel entities within the chemical space, aiming to optimize virtual screening campaigns.

First of all, we were able to develop target-specific scoring functions that showed enhanced virtual screening performance in comparison to the ChemPLP scoring function. Especially when using molecular splitter to ensure that the training and test datasets differ, the models performed similarly. This indicates that using only the protein atom contributions of the underlying scoring functions makes the model independent of the used ligand structure and focuses only on the protein interactions. This makes the production of models valuable for virtual screening approaches.

However, the focus of this work was the analysis of different decoy selection strategies. The decoy selection strategy based on random selections from other databases (ZNC, DCM) revealed behavior closest to real non-binder compounds (TRUE). So, this selection strategy appears as a suitable option for achieving useful models without the need for true inactive data. The chance that some of the decoys could in fact be active on the target seems neglectable. Since this approach needs additional computational resources to dock also all decoys, we tested diverse conformation of the active compounds that show wrong binding modes. The idea was that these wrong poses that differ from the correct binding modes provide enough information for the model to discriminate between favorable and unfavorable interactions. Although, the overall performance is clearly behind the other decoy strategies, the enrichment in virtual screening still outperforms the use of the ChemPLP scoring function. The DIV method therefore offers a computationally efficient alternative by generating different conformations from the same docking solutions, eliminating the time-intensive process of creating entirely new decoy datasets and docking the decoys. This approach potentially reduces systematic biases inherent in traditional decoy selection methods, as the generated poses represent chemically unrealistic binding modes that provide a meaningful negative control for learning valid interaction patterns. However, based on the results shown here, the performance is not as good and it depends on the available computational resources.

Nevertheless, in scenarios where molecular docking is performed in cavities with few atoms to interact, the PADIF representation might not be sufficient to differentiate actives from non-binder options. In these cases, models built with structural fingerprints can predict better than those based on protein-ligand fingerprints. Additionally, the accuracy of PADIF models is closely tied to the best pose selection and depends on optimal docking parameters. In this case, we use crystallographic poses to validate dockings and adjust the search efficiency to $200\%$, trying to guarantee that the best pose is represented by PADIF.

In conclusion, this work establishes PADIF-based machine learning models as a robust framework for virtual screening, with DCM and ZNC decoy strategies providing the optimal balance between computational efficiency and predictive accuracy. By demonstrating superior enrichment performance across diverse protein targets and rigorous validation through molecular splitters, our approach offers a practical pathway toward more effective computational drug discovery campaigns.

## Additional file

Supplementary file 1

## Data Availability

ChEMBL bioactivity data can be assessed thorugh https://www.ebi.ac.uk/chembl/. LIT-PCBA database is available in https://drugdesign.unistra.fr/LIT-PCBA/. ZINC15 can be found in https://zinc15.docking.org/. Dark Chemical Matter dataset could be find as supplementary data in the original publication webpage https://doi.org/10.1038/nchembio.1936. Finally the code and data generated could be find in this GitHub repository https://github.com/kochgroup/PADIF-wf.
