# Supplementary material for: Efficient decoy selection to improve virtual screening using machine learning models
Source: J Cheminform. 2025 Oct 30;17:165. doi: 10.1186/s13321-025-01107-z (PMC12577370; doi:10.1186/s13321-025-01107-z)
Supplement: Supplementary file 1 [file 13321_2025_1107_MOESM1_ESM.pdf]

Supporting information

Efficient Decoy Selection to Improve Virtual Screening Using Machine Learning Models

Felipe Victoria-Muñoz[1], Janosch Menke[2], Norberto Sanchez-Cruz[3], Oliver Koch[1,4*]

[1*]Institute of Pharmaceutical and Medicinal Chemistry, Universität Münster, Correnstraße 48, Munster, 48149, Nordrhein-Westfalen, Germany.

[2]Department of Computer Science and Engineering, Chalmers University of Technology, Rännvägen 4, Göteborg, 41258, Västra Götalands, Sweden.

[3]Instituto de Química, Unidad Mérida, Universidad Nacional Autónoma de México, Km 5.5 Carr. Sierra Papacal, 97302, Yucatán, México.

[4*]Center for Multiscale Theory and Computation, Universität Munster, Correnstraße 48, Munster, 48149, Nordrhein-Westfalen, Germany.

*Corresponding author(s). E-mail(s): oliver.koch@uni-muenster.de;

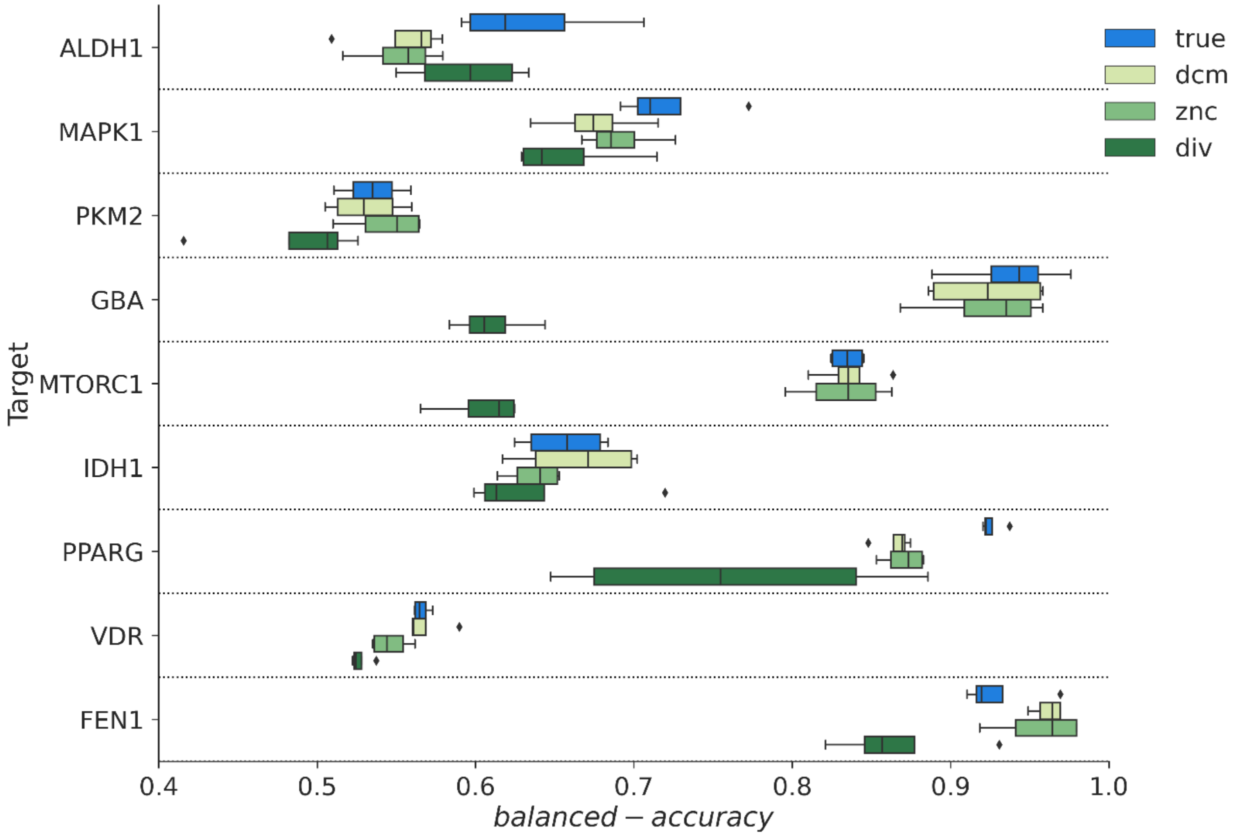

Figure S1 Balanced accuracy (BA) for models trained and tested against the fingerprint split validation set. DCM, ZNC, and diverse solution sets are represented in light green, green, and dark green, with the true dataset in blue.

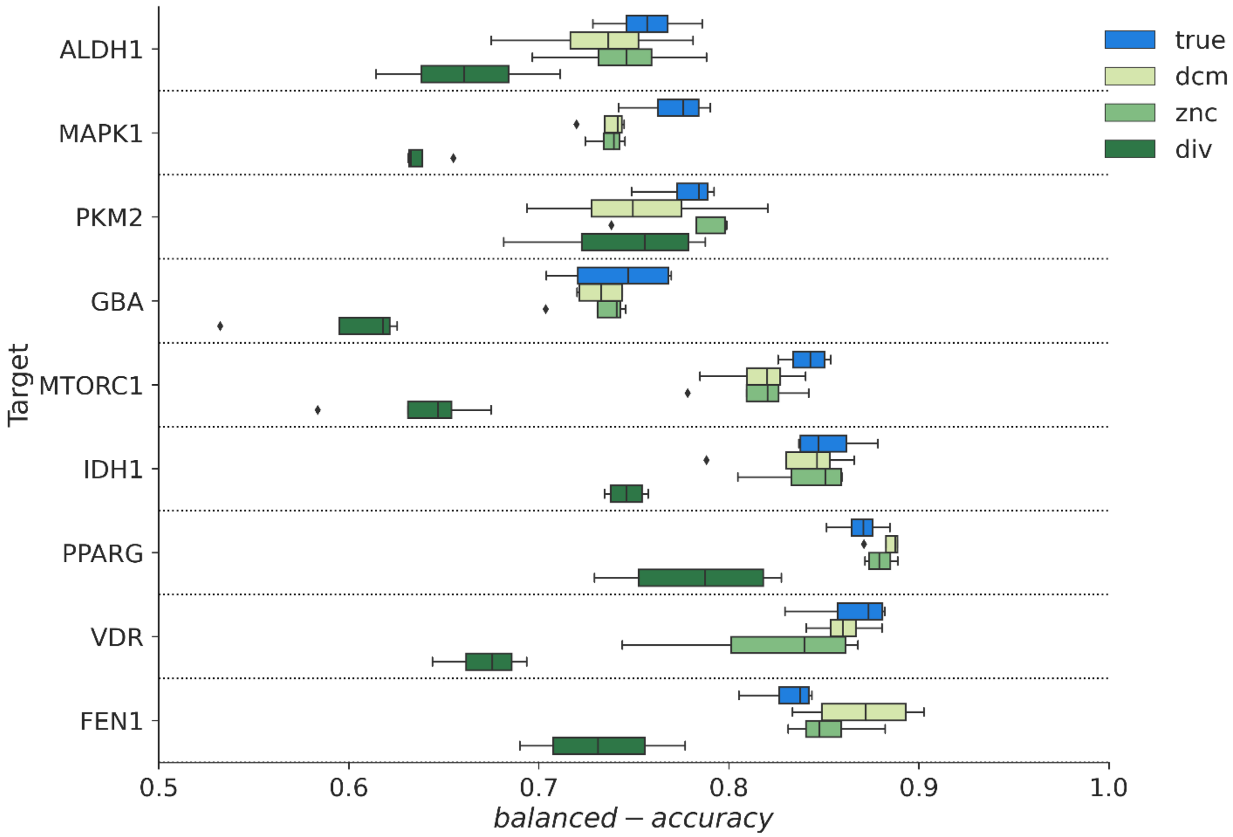

Figure S2 Balanced accuracy (BA) for models trained and tested against the scaffold split validation set. DCM, ZNC, and diverse solution sets are represented in light green, green, and dark green, with the true dataset in blue.

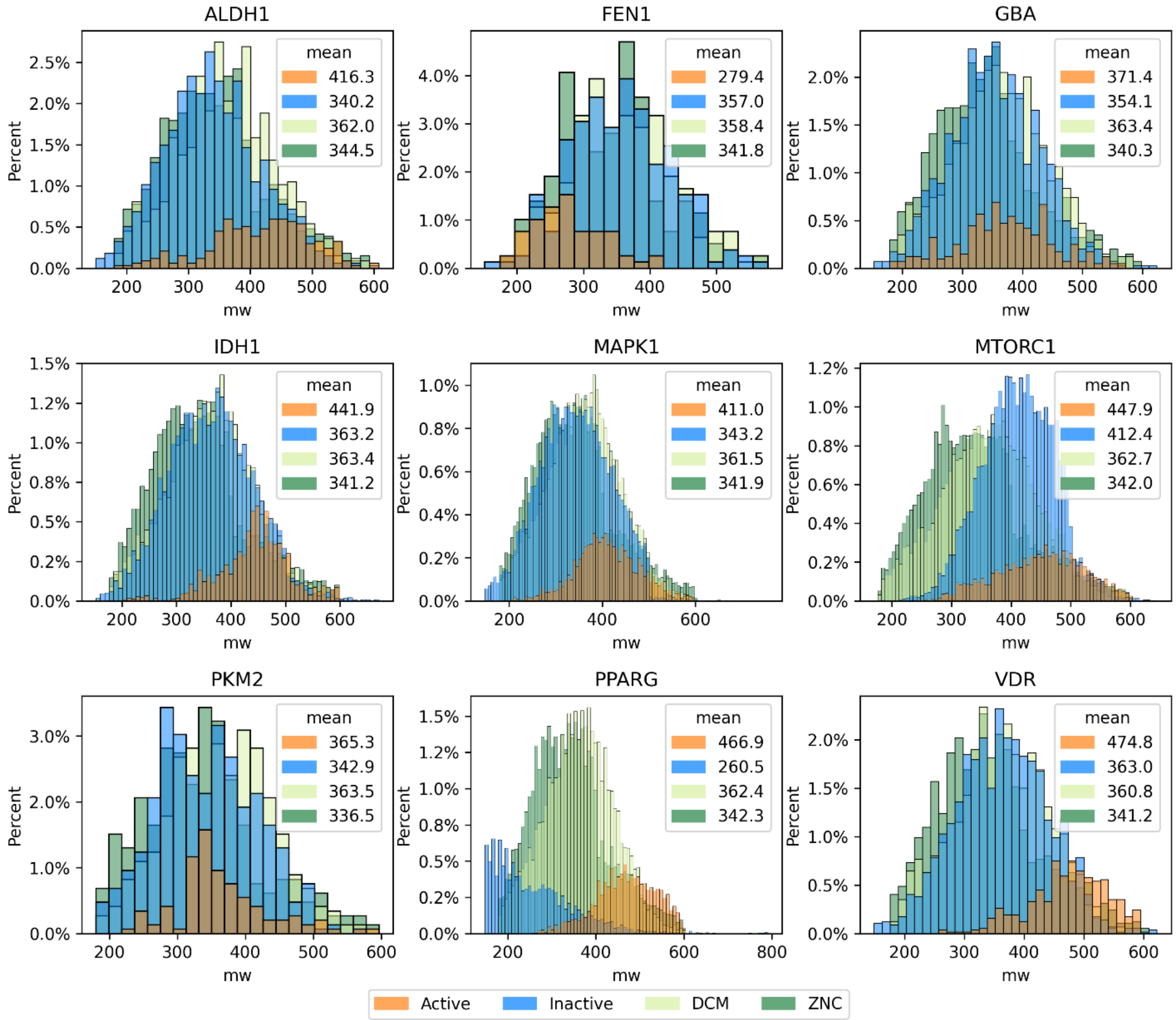

Figure S3 Histogram plot between MW and total percentage of molecules in the target sets. The legend includes the mean of ChemPLP score by category, where orange, blue, light green, and dark green represent actives, inactives, DCM, and ZNC decoys.

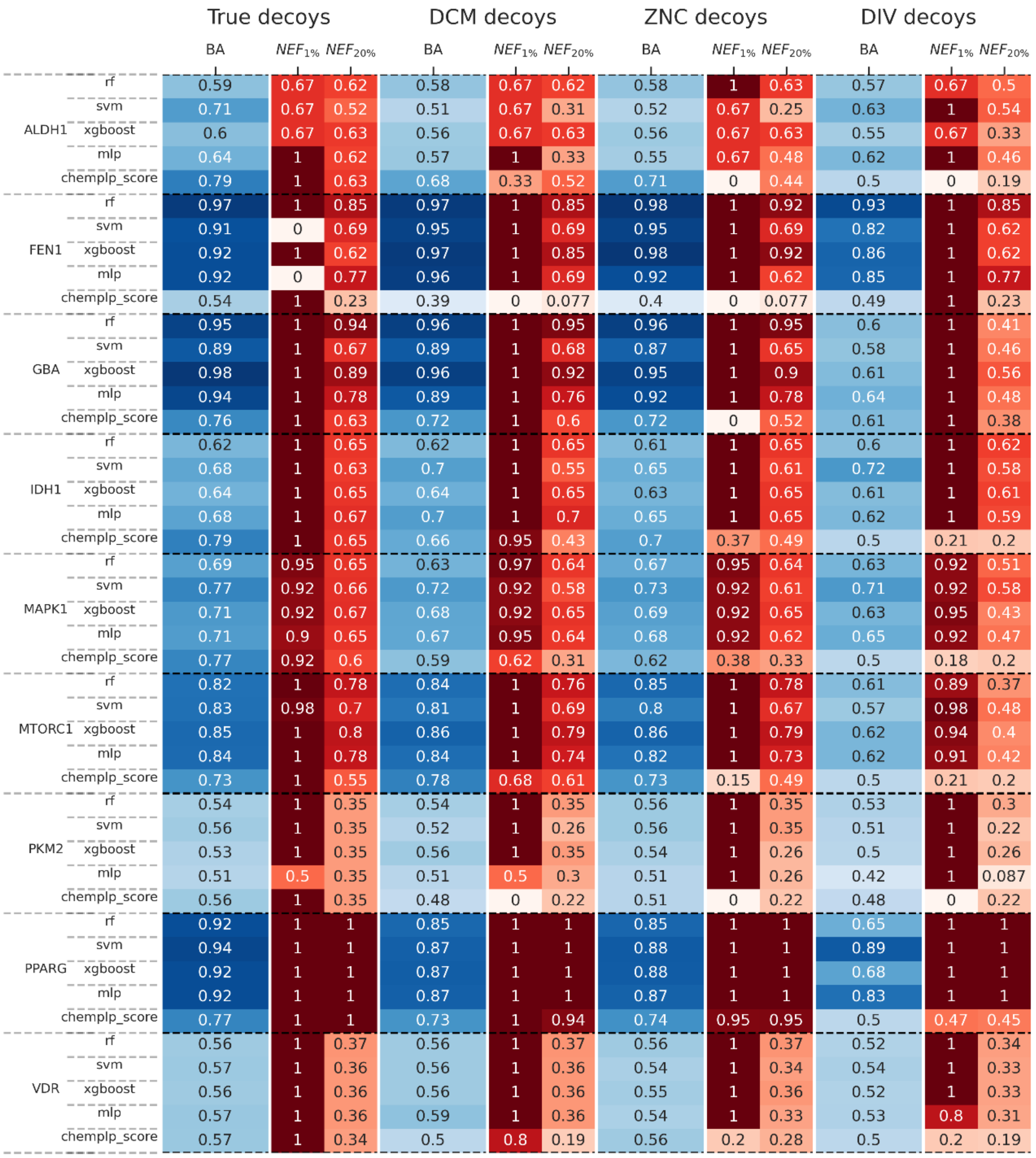

Figure S4: Heatmap displaying balanced accuracy (BA) and Normalized Enrichment Factors ($NEF_{1\%}$, $NEF_{20\%}$) for each model used (RF, SVM, XGBoost, MLP) and the ChemPLP score for each target across all decoy types in the fingerprint splitting.

Target	Model	True decoys BA	True decoys $NEF_{1\%}$	True decoys $NEF_{20\%}$	DCM decoys BA	DCM decoys $NEF_{1\%}$	DCM decoys $NEF_{20\%}$	ZNC decoys BA	ZNC decoys $NEF_{1\%}$	ZNC decoys $NEF_{20\%}$	DIV decoys BA	DIV decoys $NEF_{1\%}$	DIV decoys $NEF_{20\%}$
ALDH1	rf	0.73	1	0.6	0.74	1	0.6	0.74	1	0.62	0.71	1	0.54
	svm	0.75	1	0.62	0.68	1	0.5	0.7	1	0.56	0.61	1	0.58
	xgboost	0.76	1	0.62	0.78	1	0.65	0.79	1	0.67	0.65	1	0.44
	mlp	0.79	1	0.65	0.73	1	0.58	0.75	1	0.62	0.68	1	0.5
	chemplp_score	0.73	0.67	0.52	0.62	0.33	0.4	0.63	0.33	0.4	0.5	0.33	0.19
FEN1	rf	0.84	1	0.77	0.83	1	0.77	0.84	1	0.77	0.75	1	0.62
	svm	0.84	1	0.62	0.89	1	0.69	0.83	1	0.62	0.71	1	0.46
	xgboost	0.83	1	0.77	0.85	1	0.77	0.88	1	0.85	0.78	1	0.62
	mlp	0.81	1	0.69	0.9	1	0.85	0.85	1	0.69	0.69	0	0.54
	chemplp_score	0.59	1	0.38	0.54	1	0.23	0.54	0	0.31	0.49	0	0.23
GBA	rf	0.73	1	0.67	0.72	1	0.65	0.74	1	0.67	0.53	1	0.37
	svm	0.7	1	0.57	0.72	1	0.57	0.7	1	0.56	0.63	1	0.46
	xgboost	0.77	1	0.68	0.74	1	0.65	0.74	1	0.67	0.62	1	0.46
	mlp	0.77	1	0.7	0.74	1	0.65	0.75	1	0.68	0.62	1	0.43
	chemplp_score	0.7	1	0.54	0.65	1	0.46	0.65	0.5	0.44	0.5	0.25	0.21
IDH1	rf	0.84	1	0.76	0.84	1	0.76	0.84	1	0.76	0.74	1	0.66
	svm	0.84	0.95	0.73	0.79	1	0.67	0.8	1	0.69	0.75	0.95	0.6
	xgboost	0.86	1	0.78	0.85	1	0.78	0.86	1	0.78	0.73	1	0.63
	mlp	0.88	0.95	0.81	0.87	1	0.79	0.86	1	0.77	0.76	0.95	0.61
	chemplp_score	0.74	0.95	0.56	0.68	0.95	0.46	0.69	0.68	0.49	0.5	0.16	0.2
MAPK1	rf	0.74	0.92	0.65	0.72	0.95	0.64	0.72	0.92	0.63	0.63	0.56	0.43
	svm	0.79	0.74	0.61	0.74	0.74	0.57	0.74	0.79	0.59	0.66	0.72	0.48
	xgboost	0.77	0.95	0.67	0.74	0.92	0.66	0.74	0.92	0.63	0.63	0.72	0.43
	mlp	0.78	0.92	0.68	0.74	0.92	0.64	0.75	0.85	0.59	0.63	0.72	0.44
	chemplp_score	0.71	0.72	0.49	0.58	0.28	0.3	0.59	0.23	0.32	0.5	0.21	0.2
MTORC1	rf	0.83	0.98	0.73	0.82	0.98	0.72	0.82	0.98	0.71	0.67	0.7	0.45
	svm	0.84	0.94	0.65	0.78	0.89	0.51	0.78	0.87	0.49	0.58	0.74	0.32
	xgboost	0.85	0.96	0.75	0.84	0.94	0.76	0.84	0.94	0.73	0.65	0.77	0.36
	mlp	0.85	0.94	0.76	0.82	0.96	0.71	0.82	0.94	0.68	0.65	0.74	0.34
	chemplp_score	0.59	0.74	0.33	0.56	0.43	0.31	0.6	0.26	0.33	0.5	0.21	0.2
PKM2	rf	0.79	1	0.74	0.74	1	0.74	0.8	1	0.74	0.79	1	0.74
	svm	0.78	0.5	0.61	0.69	1	0.57	0.74	1	0.57	0.74	1	0.57
	xgboost	0.75	1	0.61	0.82	1	0.74	0.8	1	0.7	0.78	1	0.7
	mlp	0.79	1	0.7	0.76	1	0.61	0.8	1	0.7	0.68	1	0.48
	chemplp_score	0.67	0.5	0.48	0.54	0	0.3	0.54	0	0.3	0.51	0.5	0.22
PPARG	rf	0.85	1	0.99	0.87	1	0.99	0.87	1	0.99	0.73	1	0.99
	svm	0.87	1	0.99	0.89	1	0.99	0.89	1	0.99	0.83	1	0.99
	xgboost	0.87	1	0.99	0.89	1	0.99	0.88	1	0.99	0.76	1	0.98
	mlp	0.88	1	0.99	0.89	1	0.99	0.87	1	0.99	0.81	1	0.99
	chemplp_score	0.77	1	0.99	0.74	0.95	0.95	0.72	1	0.92	0.5	0.53	0.45
VDR	rf	0.88	1	0.8	0.86	1	0.78	0.87	1	0.79	0.69	1	0.69
	svm	0.83	1	0.73	0.84	1	0.75	0.74	1	0.7	0.64	1	0.55
	xgboost	0.87	1	0.79	0.88	1	0.8	0.82	1	0.74	0.67	1	0.47
	mlp	0.88	1	0.81	0.86	1	0.8	0.86	1	0.78	0.68	1	0.51
	chemplp_score	0.76	1	0.65	0.75	1	0.61	0.75	1	0.63	0.51	0.2	0.2

Figure S5: Heatmap displaying balanced accuracy (BA) and Normalized Enrichment Factors ($NEF_{1\%}$, $NEF_{20\%}$) for each model used (RF, SVM, XGBoost, MLP) and the ChemPLP score for each target across all decoy types in the scaffold splitting.

target	model	Balanced accuracy per actives-inactives ratio			
		1:2	1:4	1:8	1:10
ALDH1	rf	0.75	0.88	0.84	0.92
ALDH1	svm	0.76	0.89	0.85	0.93
ALDH1	xgboost	0.78	0.92	0.87	0.97
ALDH1	mlp	0.73	0.86	0.82	0.90
FEN1	rf	0.82	0.91	0.92	0.96
FEN1	svm	0.82	0.96	0.91	0.96
FEN1	xgboost	0.78	0.92	0.87	0.97
FEN1	mlp	0.80	0.94	0.89	0.99
GBA	rf	0.69	0.81	0.77	0.85
GBA	svm	0.69	0.81	0.77	0.85
GBA	xgboost	0.71	0.83	0.79	0.87
GBA	mlp	0.69	0.81	0.77	0.85
IDH1	rf	0.77	0.90	0.86	0.95
IDH1	svm	0.75	0.88	0.84	0.92
IDH1	xgboost	0.77	0.91	0.87	0.96
IDH1	mlp	0.79	0.93	0.88	0.98
MAPK1	rf	0.71	0.84	0.81	0.83
MAPK1	svm	0.71	0.84	0.80	0.88
MAPK1	xgboost	0.73	0.86	0.82	0.90
MAPK1	mlp	0.74	0.87	0.83	0.91
MTORC1	rf	0.75	0.88	0.84	0.92
MTORC1	svm	0.73	0.86	0.82	0.90
MTORC1	xgboost	0.77	0.91	0.87	0.96
MTORC1	mlp	0.77	0.90	0.86	0.95
PKM2	rf	0.65	0.77	0.73	0.81
PKM2	svm	0.53	0.62	0.59	0.65
PKM2	xgboost	0.65	0.76	0.72	0.80
PKM2	mlp	0.61	0.72	0.68	0.76
PPARG	rf	0.80	0.94	0.89	0.99
PPARG	svm	0.81	0.95	0.90	0.91
PPARG	xgboost	0.82	0.96	0.91	0.90
PPARG	mlp	0.80	0.94	0.89	0.99

Table S1: Balanced accuracy per models trained with variations in the active and inactive ratio

target	actives in train	actives in test	total actives	chembl mols
Aldehyde dehydrogenase 1A1	232	26	258	281
Beta glucocerebrosidase	280	32	312	342
Flap endonuclease 1	54	7	61	61
Isocitrate dehydrogenase NADP cytoplasmic	1637	182	1819	1825
MAP kinase ERK2	3462	385	3847	3853
Peroxisome proliferator activated receptor gamma	3811	424	4235	4328
Pyruvate kinase isozymes M1-M2	100	12	112	113
Serine threonine-protein kinase mTOR	4171	464	4635	4658
Vitamin D receptor	399	45	444	444

Table S2: Actives per Train, Test sets and total used for each target.
